# Adeno-tonsillectomy surgery in a joint aid effort: a feasible solution?

**DOI:** 10.1016/S1808-8694(15)30096-3

**Published:** 2015-10-19

**Authors:** Marcos Luiz Antunes, Ricardo Frazatto, Eduardo Kosugi Macoto, Fernando Mirage Vieira, Fernando Kaoru Yonamine

**Affiliations:** a Master’s degree and Doctoral degree, UNIFESP. Professor of the ABC medical college. Coordinator of the Otorhinolaryngology unit of the Diadema State Hospital.; b Master’s degree in otorhinolaryngology, UNIFESP. Physician of the Diadema State Hospital.; c Otorhinolaryngologist, UNIFESP. Master’s degree student in the Otorhinolaryngology and Head & Neck Surgery Department, UNIFESP.; d Physician, UNIFESP. 3rd year resident in the Otorhinolaryngology and Head & Neck Surgery Department, UNIFESP.; e Physician, UNIFESP. 2nd year resident in the Otorhinolaryngology and Head & Neck Surgery Department, UNIFESP. UNIFESP - Diadema State Hospital.

**Keywords:** tonsil, pharyngeal, palatine, tonsil

## Abstract

Public hospitals in Brazil are under capacity for adenotonsillectomies, resulting in a growing waiting line. Otolaryngologists are used to these lines, since they understand that this problem is under govern responsibility. For this reason we believe that joint aid efforts to carry out adenotonsillectomies are justified.

**Aim:**

To standardize the organization of adenotonsillectomies in joint aid efforts, its effectiveness and feasibility for public hospitals, and to compare the incidence of post-operative hemorrhage in joint aid effort surgery with that of regular surgeriy. **Methods:**A clinical case-control prospective study of adenotonsillectomies done in joint aid efforts was done from September 2004 to June 2006 at the Diadema State Hospital. An analysis was made of the multiprofessional staff involved in this process, and a comparison was made of the incidence of hemorrhage in joint aid efforts and after regular surgery.

**Results:**

22 joint aid effort events for adenotonsillectomies were done during the period mentioned above (339 surgeries), an average 15.4 surgeries per event. The rate of postoperative hemorrhage requiring surgical revision was 1.48%(5/339), which did not differ statistically from the case-control group (1.37% - 5/364).

**Conclusion:**

We were able to standardize the results of adenotonsillectomies done in a joint aid effort to the parameters that are considered as safe. This may reduce the waiting line for this procedure. The difference in the incidence of postoperative hemorrhage in the joint aid effort and regular surgery was not statistically significant.

## INTRODUCTION

Tonsillectomies and adenoidectomies are among the most frequent surgical procedures in the daily practice of otorhinolaryngologists, both in public and private medical settings. Indications for these procedures have changed little. Upper airway obstruction caused by increased tonsillar or adenoid volume is still the main indication, particularly in children. Other indications are recurring tonsillitis, peritonsillar abscesses and systemic diseases that may be worsened by tonsillitis. A relative indication is halitosis in caseous tonsillitis.

Not all hospitals have otorhinolaryngologists; these professionals are lacking in public health services. Those hospitals that do have these specialists and the basic conditions for undertaking otorhinolaryngological surgical procedures are under great demand, resulting in a long waiting list that grows in a geometric progression. There is a repressed demand and a long waiting time for patients that require these procedures.

Surgeons working in a public health unit become used to the waiting lines, possibly believing that the problem belongs to the State; the Brazilian constitution states that “health is a civil right and a State duty” (article 196 of the Brazilian Constitution).^1^ This issue is not discussed in medical and academic debates.

The otorhinolaryngology specialist, who is closer to the patient, and has diagnosed the case and indicated surgery, faces a serious public health issue. This problem may lead to impaired child growth, tube obstruction leading to chronic otitis media, swallowing difficulties, reduced olfaction and sense of taste, speech disorders, altered facial growth and complications due to sleep apnea. Physicians should not turn their backs on this issue and neglect the anguish and pain of parents who frequently spend their nights awake fearing that their children, in their own words, will “stop breathing.” Many of these parents wander from hospital to hospital in an attempt to speed up the whole process, usually without success, given that this is a public health issue. It is a true marathon for parents after having already faced a waiting line for the first visit to a specialist.

Some hospital simply refuse new cases, establishing a specific number of patients in the waiting line not to increase the waiting time for surgery.^1^ This appears to solve the problem for the specific hospital, but not for the population at large.

The idea of a joint effort is that a larger number of surgeries may be done in a short time period to increase the offer and to meet the demand for surgery. A joint effort for adenoidectomies and tonsillectomies requires thinking about possible complications. A significant number of surgeries done in a short time period may increase the possibility of surgical complications, especially hemorrhage.

Given this background, we believe it is essential to undertake joint efforts or task forces for adenoidectomies and tonsillectomies. Our work was to carry forward this practice in a public hospital, with the following aims:

1. to standardize the organization process for a task force or joint effort, and to check whether this would be possible in public hospitals that already are under significant demand for these procedures;
2. to compare the rate of the most frequent postoperative complication (hemorrhage) in joint effort procedures and in routinely done surgery.

## MATERIAL AND METHODS

We conducted a clinical prospective cohort trial between August 2004 and June 2006.

The trial took place in the Diadema State Hospital, a public hospital jointly managed by the SPDM (Paulista Medical Society) and the UNIFESP (Sao Paulo Federal University). The hospital provides free treatment for patients and teaching for residents of the Otorhinolaryngology and Head & Neck Surgery Department, UNIFESP.

An initial meeting with the coordinator of the otorhinolaryngology department and the clinical director of the hospital was held to discuss the problem and to assess the feasibility of a joint effort for adenoidectomies and tonsillectomies.

The next step was to assess the waiting list at the time this project was started, on August 2004. At that time, the list was on paper by order of arrival to the otorhinolaryngology unit. The list was fed into a computer and three backup copies were sent to different computers and to floppy disks. A social worker updated the list by telephoning to each patient; patients that had already been operated in other hospitals or units, those that no longer lived in the state of Sao Paulo and patients with outdated telephone numbers were removed from the list. At this point, the list had been reduced from the original 850 patients to 590 patients. On average 3 patients were entered into the list every day, about 60 patients a month. We routinely perform 36 adenoidectomies and/or tonsillectomies every month, so that the excess demand is 24 patients a month (288/year), not including those already on the waiting list.

Patients were then asked to visit the otorhinolaryngology unit at a rate of 5 new cases per outpatient unit period to review the indication for surgery, the clinical status and the preoperative exams. These exams included a complete blood count and coagulation tests; any altered tests were repeated. If the test alteration persisted, the patient was referred to the hematology unit for assessment. Following confirmation of the clinical status and if preoperative exams were within normal limits, patients entered the joint effort waiting list. The joint effort was for 18 surgeries once a month on a Saturday, for children aged between 0 and 14 years. When the number of patients reached the limit, the next patient was placed on the next joint effort waiting list or on the routine surgery waiting list, whichever was first. The task force included three otorhinolaryngologists from the hospital staff and three anesthesiologists, forming teams in the three surgical theaters of the hospital, each operating six cases throughout the day. Surgeons and anesthesiologists were paid as for a 12-hour on duty period each. Nursing team hours of work were maintained in an “overtime bank”.

The surgical team also included a surgical nurse, three operating room technicians, an anesthesiology assistant and two support technicians for post-anesthetic recovery, organization of the operating room and control of equipment and materials. Six surgical kits were available in the hospital and surgeons brought another three surgical kits, as the hospital did not have a flash autoclave.

Patients were admitted the night before surgery; the preoperative prescription included venoclysis, endovenous saline and no food or liquids after 10 p.m. Patients were called for surgery by increasing order of age. The surgeon and the anesthesiologist reassessed the patient, talked to parents about postoperative care, and clarified their eventual doubts. Preanesthetic medication was midazolam. Surgery was done under general anesthesia; the surgical technique was tonsillar dissection and adenoid curettage using Beckmann adenoid curettes. Pharyngeal tonsillar hemostasis was done with gauze anchored to the rhinopharynx for 10 minutes; palatine tonsillar hemostasis was done with 2.0 plain catgut sutures.

After surgery, patients were taken to the recovery room to be monitored for hemorrhage. Patients were discharged after at least 40 minutes if there was no bleeding and the patient was stable. Patients remained in the hospital ward receiving endovenous glucose and saline, an antibiotic and an analgesic. Patients were discharged on the following day (Sunday) by two otorhinolaryngologists of the team (each receive payment equivalent to three hours of work). Patients returned for follow-up after five to seven days; a second visit was scheduled for one month after surgery. Parents were asked to bring the child at any time if needed.

We compared the waiting line before this study was undertaken with the current situation, after the joint effort, to quantify the efficiency of the task force in reducing the waiting list for surgery.

We used a retrospective study of similar surgeries done between September 1999 and May 2001 in the Diadema State Hospital and the Pirajussara General Hospital, both managed by SPDM/UNIFESP and therefore similar in structure and operation. This study served for comparison purposes to assess postoperative hemorrhage in our cases.2 Statistical analysis was done using the chi-square test and the difference between two proportions test, at a 5% significance level.

## RESULTS

The trial period (August 2004 to June 2006) included 22 joint efforts; the first one took place on September 2004. There were 339 surgeries done in the full joint effort, distributed according to the type of procedure (Chart 1).

**Graph 1:**
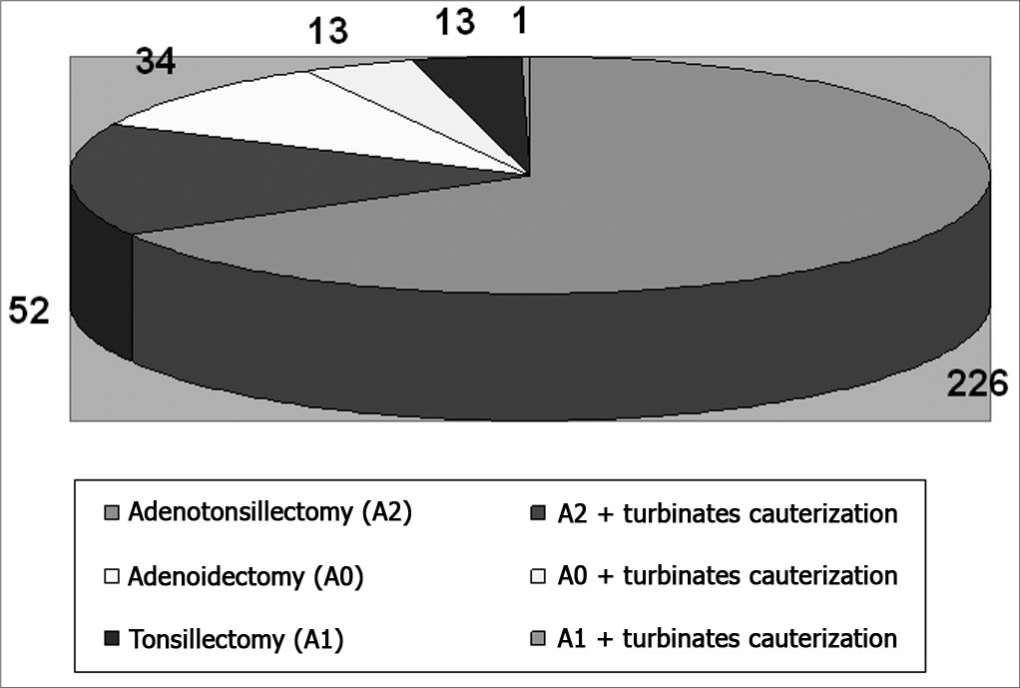
Patient distribution based on the type of surgery performed

The mean number of surgeries per day was 15.4. The age of patients varied from 2 to 14 years; there were 175 females and 164 males. The number of surgeries that were not done for medical reasons or because the patient did not appear (personal problems or patients with another disease, mostly upper airway infection) was 57. The mean surgical time throughout the joint effort period was 32.46 minutes (Chart 2). The mean duration of each joint effort day was 8 hours and 13 minutes.

**Graph 2:**
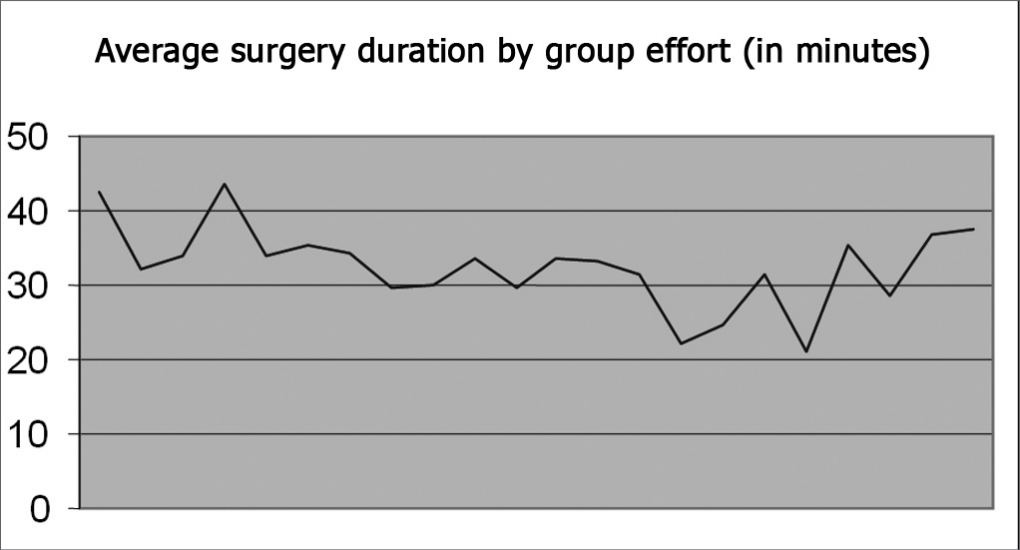
Patient distribution based on average time spent per surgery

Each procedure required a mean of two catgut suture packets, including revision surgical procedures.

There were five postoperative hemorrhages in the first 24 hours after surgery, all after adenotonsillectomies. These patients were reoperated for control of bleeding, which in all cases was on the adenoidectomy site. One case required packing of the rhinopharynx; the others were controlled by optic fiber guided electrocauterization.

The patient that required packing had normal prothrombin activity (PA) and an altered activated partial thromboplastin time (APTT) which was 47.1 seconds and a patient/normal ratio of 1.23. Surgery had been authorized after a preoperative consultation with a hematologist. Anterior and posterior packing was removed with no further bleeding.

A comparison of the postoperative hemorrhage rate between the joint effort group (5/339 cases) and the control group (5/364 cases) using the chi-square test revealed a p value of 1.0000. The difference between two proportions test revealed a p value of 0.9021. Both differences are not statistically significant (Table 1).

**Table 1:** Comparison of task force and routine (control group) surgeries with and with no postoperative hemorrhage.

Surgeries	Surgical procedure			
	Task force		Routine surgery	
	N	%	N	%
Surgeries with no hemorrhage	334			
	98,52	359	98,63	
Surgeries with hemorrhage	05	1,48	05	1,37

Total	339	100	364	100

**Key:**

N = number

Chi-square test (p=1,0000)

Difference between two proportions test (p=0.9021)

The waiting list fell from 590 patients (initial number) to about 340 patients (after the joint effort), a reduction of about 250 patients.

## DISCUSSION

We found that the waiting list has to be defined more precisely in order to quantify the true problem. A carefully documented registry of patients and support from a social worker, a professional that is trained for this function, is essential in our view.

The nest step was to make the hospital directors aware of the problem and to demonstrate the feasibility of joint efforts in term of available professionals, material resources and funding. Many hospitals may question this point, as joint efforts require paying extra wages and materials. Law number 1196 of 17.08.01 D.O.U and number 159-E of 20.08.01 foresees funding for such joint efforts in otorhinolaryngological surgery.^3^ Vascular surgeons are also undertaking joint efforts for varicose vein surgery, and some authors have assessed the cost of admitting these patients relative to funding provided by the Ministry of Health for these joint efforts.^4^

Other medical specialties are used to task forces for surgical procedures,^5^, ^6^ including ophthalmology - with its cataract task forces - which in 2003 met 100% of the demand for public health cataract surgery.^6^ These efforts have encouraged us to continue with our joint efforts and to inspire other physicians to do the same in other public hospitals. Our waiting lists are far longer than most of those in other medical specialties. Surgery causes a significantly positive impact on the quality of life of our pediatric patients; their loss when not operated for months or years is immeasurable. According to the Brazilian bylaws for children and adolescents, these age groups should always be prioritized in public hospitals and in public health policies.^1^

We believe that 18 surgeries is an adequate number for each joint effort day. The mean number of surgeries done in each joint effort day was 15.4 procedures. Adenoidectomy with or without tonsillectomy is stressful both for the anesthesiologist and the surgeon. We deal with upper airway surgery in a pediatric patient, where the endotracheal tube needs to be displaced during the procedure. Furthermore, the most feared complication - postoperative hemorrhage - is not always easily controlled, apart from bringing additional difficulties for the anesthesiologist, who is required to access an airway with blood and clots in the event of a revision surgery. We found that bleeding in all cases that required a revision surgery was on the adenoidectomy site; hemostasis of this region is difficult, and required an optic fiber. It appears to us that quality work at a lower risk for patients is possible when each surgeon performs six surgeries per day in a joint effort. The mean duration of each joint effort day was 8 hours and 13 minutes. After this period, the team was tired, and was required to be prepared for a possible revision surgery due to hemorrhage. At the end of each day, sufficient surgical material had to be available for such a procedure.

After planning the number of surgeries and comparing the joint effort work and routine surgery in the hospital, we found that the incidence of postoperative hemorrhage was similar, with no statistically significant difference. The incidence is also within the parameters established in the literature7 and even slightly lower than that in routine surgery.^8^

The hospital directors had feared surgical and anesthesiological complications in joint efforts such as these. In order to reduce complications to similar levels as those in routine surgery, we took into account the fact that the team would be tired after a specific number of procedures, and that it could be required to reintervene in the event of complications. We believe that the number of potentially fatal complications would increase significantly if the number of programmed surgeries were increased.

The rate of hemorrhage in the first 24 hours and in the first week postoperatively in our joint effort work was similar to that of routine surgery (1.48%). The need for revision surgeries to deal with complications was similar to the control group (1.37%), with no statistical difference. The study that we used as our control group was done in two public hospitals (the Diadema State Hospital and the Pirajussara General Hospital). In this study, there were 364 elective surgeries, done by medical residents of the UNIFESP under guidance from their tutors, in the routine hospital timetable. Two to three surgeries were done per day; if there were complications, the tutor performed the revision surgery.^2^ Based on this information, we believe that fully trained surgeons should perform surgery in joint efforts; if residents carry out the procedure, they should be constantly monitored by a tutor to provide support in there are complications.

We found the comparison between our joint effort work and the abovementioned paper appropriate, as surgical techniques were the same and the two series are similar. Both had a very satisfactory hemorrhage rate, particularly when we compared these trials with a third study within the Brazilian context, where the rate of immediate postoperative bleeding was 7.48%.^8^

Reduced waiting lines, the improved quality of life of our patients, the satisfaction of their parents in having obtained a solution for their children’s health problem, and the low rate of surgical complications should help to encourage our otorhinolaryngological colleagues to undertake similar joint efforts. The issue of waiting lines for adenotonsillectomies is a public health problem with a feasible solution: joint efforts!

## CONCLUSION

1. We were able to standardize the joint effort for adenotonsillectomy surgery within what we consider the safest parameters.
2. The incidence of postoperative hemorrhage was not statistically different between the joint effort and the routine surgeries.
